# Rapid Determination of Antimicrobial Susceptibility by Stimulated Raman Scattering Imaging of D_2_O Metabolic Incorporation in a Single Bacterium

**DOI:** 10.1002/advs.202001452

**Published:** 2020-08-16

**Authors:** Meng Zhang, Weili Hong, Nader S. Abutaleb, Junjie Li, Pu‐Ting Dong, Cheng Zong, Pu Wang, Mohamed N. Seleem, Ji‐Xin Cheng

**Affiliations:** ^1^ Department of Electrical and Computer Engineering Boston University Boston MA 02215 USA; ^2^ Boston University Photonics Center Boston MA 02215 USA; ^3^ Department of Comparative Pathobiology Purdue University West Lafayette IN 47907 USA; ^4^ Department of Biomedical Engineering Boston University Boston MA 02215 USA; ^5^ Vibronix Inc. West Lafayette IN 47906 USA; ^6^ Purdue Institute of Inflammation Immunology, and Infectious Disease West Lafayette IN 47907 USA; ^7^ Department of Chemistry Boston University Boston MA 02215 USA

**Keywords:** antimicrobial susceptibility testing, bacteria, isotope probing, single‐cell imaging, stimulated Raman scattering

## Abstract

Rapid antimicrobial susceptibility testing (AST) is urgently needed for treating infections with appropriate antibiotics and slowing down the emergence of antibiotic‐resistant bacteria. Here, a phenotypic platform that rapidly produces AST results by femtosecond stimulated Raman scattering imaging of deuterium oxide (D_2_O) metabolism is reported. Metabolic incorporation of D_2_O into biomass in a single bacterium and the metabolic response to antibiotics are probed in as short as 10 min after culture in 70% D_2_O medium, the fastest among current technologies. Single‐cell metabolism inactivation concentration (SC‐MIC) is obtained in less than 2.5 h from colony to results. The SC‐MIC results of 37 sets of bacterial isolate samples, which include 8 major bacterial species and 14 different antibiotics often encountered in clinic, are validated by standard minimal inhibitory concentration blindly measured via broth microdilution. Toward clinical translation, stimulated Raman scattering imaging of D_2_O metabolic incorporation and SC‐MIC determination after 1 h antibiotic treatment and 30 min mixture of D_2_O and antibiotics incubation of bacteria in urine or whole blood is demonstrated.

## Graphical abstract

A phenotypic platform that rapidly produces antimicrobial susceptibility testing results by femtosecond stimulated Raman scattering imaging of D_2_O metabolism is reported. Metabolic incorporation of D_2_O into biomass in a single bacterium and the metabolic response to antibiotics are probed. Single‐cell metabolism inactivation concentration is obtained in less than 2.5 h from colony to results.

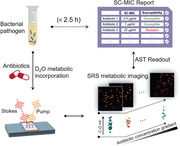

## 1 Introduction

Antimicrobial resistance has become a growing public threat, causing nearly 1 million related mortality each year globally.^[^ ^1^ ^]^ It was estimated that by 2050 antimicrobial resistance will cause 10 million deaths and $100 trillion global production loss if no action is taken.^[^ ^1^, ^2^ ^]^ To combat this crisis, rapid antimicrobial susceptibility testing (AST) is essential to slow down the emergence of antimicrobial resistance and consequently reduce the deaths caused by drug‐resistant infections.^[^ ^3^ ^]^ The gold standard for AST is conducted by disk diffusion or broth dilution methods and used to determine whether the bacteria are susceptible, intermediate or resistant to antimicrobial agents tested.^[^ ^4^ ^]^ After 16–24 h incubation, a minimal inhibitory concentration (MIC) value is read as complete growth inhibition through visual inspection. Current culture‐based phenotypic method for AST is too slow to guide immediate decision for infectious disease treatment.^[^ ^5^ ^]^ For clinical samples, it usually takes at least 24 h for bacterial preincubation and at least additional 16 h for AST, which is time‐consuming and leads to the emergence and spread of antimicrobial resistance.^[^ ^6^ ^]^

Genotypic methods, such as polymerase chain reaction (PCR)‐based techniques,^[^ ^7^ ^]^ do not rely on culturing and provide faster results, but they only target specific known genetic sequences with resistance and thus, are not generally applicable to different bacterial species or mechanisms of resistance, nor providing MIC results for therapy decisions.^[^ ^8^ ^]^

To overcome these limitations, novel phenotypic methods for rapid AST are under development,^[^ ^9^ ^]^ including microfluidic devices that increase the detection sensitivity by confining the sample in a small area,^[^ ^10^ ^]^ monitoring bacterial growth or morphological changes at single cell level,^[^ ^10^, ^11^ ^]^ phenotypic AST quantifying the nucleic acids copy number,^[^ ^12^ ^]^ and Raman spectroscopy that probes the chemical content inside a bacterium.^[^ ^13^ ^]^ While these methods reduce the time for AST, most of them only work for bacterial isolates and are still based on culture of the target pathogen, remaining too slow to support immediate clinical treatment decisions. Only a few strategies can directly work on challenging clinical samples. Specifically, a method using nanoliter droplet arrays with the fluorescence dye resazurin indicating cell viability is able to perform AST directly on bacteria harvested from clinical urine samples.^[^ ^8a^ ^]^ Digital real‐time loop‐mediated isothermal amplification is used with microfluidic devices to determine the phenotypic antibiotic susceptibility of bacteria in clinical urine samples in less than 30 min.^[^ ^12b^ ^]^ Single‐cell Raman spectroscopy on metabolic response to antibiotic,^[^ ^14^ ^]^ large volume light scattering microscopy,^[^ ^15^ ^]^ and a fidget spinner for concentrating pathogens^[^ ^16^ ^]^ were directly applied for clinical urine samples. However, these methods neither determined the MIC values nor tested blood samples. A direct method based on microscopic image analysis can determine the AST and provide MIC values from positive blood cultures.^[^ ^17^ ^]^ Yet, it relies on bacterial growth and takes at least 6 h to perform AST.

Inside a cell, NADPH is ubiquitously used for biomolecular synthesis.^[^ ^18^ ^]^ Based on rapid enzyme‐catalyzed exchange between the redox‐active H atom in NADPH and the D atom in deuterium oxide (D_2_O), so‐called heavy water, cellular metabolic activity can be probed via monitoring the intracellular conversion of D_2_O into C–D bonds of the biomolecules. Raman spectroscopy relies upon inelastic scattering of light, indicated by a shift in the energy of incident photons. The shift in energy provides the native fingerprint vibrational information of a molecule determined by its structure and environment. As a versatile analytical tool providing chemical bond information of biomolecules, spontaneous Raman spectroscopy has been used to determine antimicrobial susceptibility by detecting de novo synthesized C–D bonds in biosystems incubated in D_2_O‐containing medium.^[^ ^14^, ^19^ ^]^ However, the sensitivity of Raman spectroscopy is intrinsically low due to extremely small Raman scattering cross‐sections, which does not allow rapid and high‐throughput AST. By spontaneous Raman measurement, it usually takes ≈10 min (30 s per spectrum) to acquire Raman spectra of 20 individual bacteria. Thus, to determine MIC via ten concentrations of one antibiotic, the total Raman measurement time per strain would be 100 min. Thus, it would need at least 17 h to determine MIC of ten antibiotics for one strain. In contrast, by focusing the excitation energy on a target Raman band, coherent Raman microscopy based on either coherent anti‐Stokes Raman scattering (CARS) or stimulated Raman scattering (SRS) provides orders‐of‐magnitude signal enhancement, thereby enabling high‐speed chemical imaging of single cells.^[^ ^20^ ^]^ For broad Raman bands such as C–H and C–D stretch vibrations, femtosecond SRS further boosts the signal level.^[^ ^21^ ^]^

Here, we report a rapid phenotypic platform that can determine the susceptibility of bacteria in urine and whole blood by femtosecond SRS imaging of D_2_O metabolism in a single bacterium. Harnessing the high sensitivity of femtosecond SRS imaging, D_2_O metabolic incorporation inside a single bacterium in the presence of antibiotics is probed in as fast as 10 min. Unlike spontaneous Raman spectroscopy, a C–D SRS image covering tens of bacteria is recorded in ≈1 s. In the presence of antibiotics, a single‐cell metabolism inactivation concentration (SC‐MIC) is determined in less than 2.5 h from colony to results. Comparison of SC‐MIC results with conventional MIC results among 37 sets of samples, including eight major bacterial species and 14 different antibiotics often encountered in clinic, yields a category agreement of 94.6% and 5.4% minor error. Moreover, our method is able to determine the metabolic activity and susceptibility of bacteria in either urine or whole blood environment, which opens the opportunity for rapid single‐cell phenotypic AST in clinic.

## 2 Results

### 2.1 SRS Imaging of D_2_O Metabolic Incorporation in a Single Bacterium

**Figure 1:**
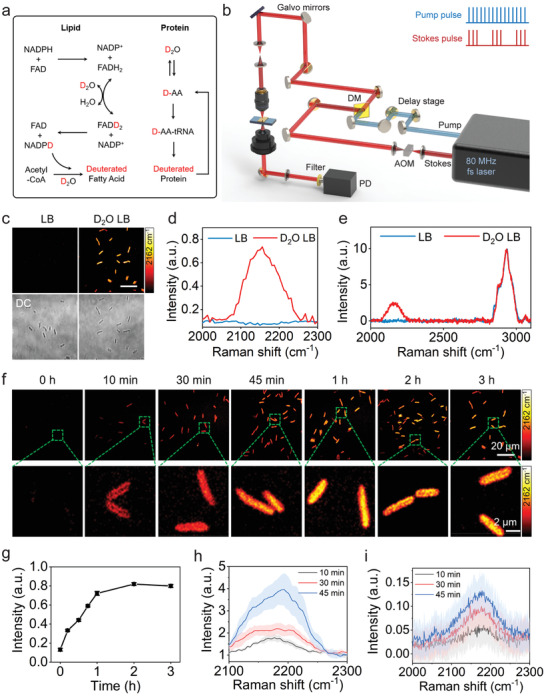
SRS imaging of D_2_O metabolic incorporation in a single bacterium. a) Scheme for D_2_O labeling of lipid and protein. b) Schematic illustration of SRS setup. AOM: acousto‐optic modulation. DM: dichroic mirror. PD: photodiode. c) SRS and corresponding transmission images of *P. aeruginosa* after culture in normal and D_2_O‐containing medium for 3 h. Scale bar: 20 µm. SRS spectra d) and spontaneous Raman spectra e) of *P. aeruginosa* after culture in normal and D_2_O containing LB medium for 3 h. f) Time‐lapse SRS imaging of *P. aeruginosa* after culture in D_2_O containing medium. g) Average C–D intensity plot over time for bacteria in (f) with *N* ≥ 10 per group. Hyperspectral SRS (*N* = 5) h) and spontaneous Raman (*N* = 20) i) spectra at the C–D signature region, corresponding to the time‐lapse D_2_O incorporation of *P. aeruginosa* after 10, 30, and 45 min culture. Error bars represent the standard error of the mean (SEM).

In cells, flavin enzymes catalyze the H–D exchange between water and NADPH's redox active hydrogen in D_2_O containing media. The deuterium labeled NADPH mediates fatty acid synthesis reaction with D_2_O incorporation, resulting in the deuterated fatty acids production. Biosynthetic pathway of deuterated proteins is through introducing deuterium atoms from D_2_O into reactions of amino acids (AAs) (**Figure** **1**a).^[^ ^18^, ^22^ ^]^ The schematic of our SRS microscope is shown in Figure 1b. In brief, spatially and temporally overlapped pump and Stokes pulses are tuned to match the vibrational frequency of Raman‐active modes. The SRS signal appears as an intensity gain in the Stokes beam and an intensity loss in the pump beam, which is extracted through a lock‐in amplifier. Stimulated Raman loss is measured, in which most excitation power is in the 1040 nm Stokes beam having a high cellular damage threshold. The carbon–deuterium (C–D) vibrational band, which is spectrally differentiated from endogenous Raman bands, is selectively detected with SRS using either chirped or nonchirped femtosecond laser pulses. Previously,^[^ ^23^ ^]^ we used chirped femtosecond pulses for hyperspectral SRS imaging of C–D bonds in bacteria. To enhance the detection sensitivity and speed up the imaging process, we applied nonchirping femtosecond pulses and increased the signal to noise ratio by ≈5‐folds (Figure S4, Supporting Information). With femtosecond SRS, C–D signals from all bacteria in the field of view could be obtained at a speed of ≈1.2 s per image of 200 × 200 pixels, at a pixel dwell time of 30 µs. Therefore, femtosecond SRS imaging enables high‐speed study of D_2_O incorporation at single bacterium level.

We then examined the toxicity of D_2_O to bacterial cells. Unlike mammalian cells, bacteria are much more resistant to D_2_O toxicity. Our experiments showed that 70% D_2_O concentration did not cause severe growth inhibition (Figure S1, Supporting Information). Thus, we chose 70% D_2_O containing medium to culture bacteria in the following studies. By tuning the Raman shift to C–D vibration at ≈2162 cm^−1^, strong signals were observed at individual bacteria after culture in D_2_O containing medium for 1 h (Figure 1c; Figure S2, Supporting Information). As a control, no C–D signal was observed for bacteria cultured in normal medium (Figure 1c; Figure S2, Supporting Information). These results were further confirmed by SRS spectra (Figure 1d) obtained through temporal tuning of chirped pump and Stokes pulses, and spontaneous Raman spectra (Figure 1e), both showing a broad peak (from 2070 to 2250 cm^−1^) at C–D vibration only for bacteria cultured in D_2_O containing medium. Therefore, SRS imaging at C–D vibrational region provides a good means to monitor D_2_O incorporation in a single bacterium.

To verify metabolic D_2_O incorporation in bacteria, we measured the cellular metabolic activity kinetics under different incubation conditions (Figure S3, Supporting Information). As depicted in the SRS images, the live *Pseudomonas aeruginosa* cells, cultured in D_2_O containing medium at 37 °C, had high metabolic activities and exhibited increasingly stronger C–D intensities with increased incubation time. In contrast, neither live *P. aeruginosa* incubated at 4 °C, nor formalin‐fixed *P. aeruginosa*, incubated at 37 °C, showed observable C–D signals because of the metabolic activity inhibition. Our findings confirm that C–H bonds are unlikely to undergo abiotic H–D exchange. Instead, cellular metabolic activity directly relates to D_2_O incorporation, which is reflected by biochemical transformation of forming C–D bonds in newly synthesized biomolecules.^[^ ^18^, ^24^ ^]^

Next, we investigated whether SRS imaging could resolve the fast D_2_O incorporation in biomolecule synthesis spatially and temporally. Time‐lapse SRS images (Figure 1f) and statistical analysis (Figure 1g) showed that the average intensity of C–D signals in *P. aeruginosa* increases with time and saturates at ≈2 h. With the enhanced detection sensitivity, C–D signals in individual *P. aeruginosa* can be observed after culture in as short as 10 min, which is shorter than the generation time of *P. aeruginosa* (24 to 27 min).^[^ ^25^ ^]^ These results showed that the D_2_O incorporation of bacteria can be detected by SRS within one cell cycle.

With sub‐micron spatial resolution, we further observed the differential distribution of C–D signals in 10 min, 30 min and longer culture time (Figure 1f). After 10 min culture, a stronger signal was observed at cell periphery than that at the center of bacteria (Figure 1f). In contrast, with 30 min and longer culture times, the signal intensity was stronger at the cell center than that at the cell periphery (Figure 1f). The hyperspectral SRS spectra (Figure 1h) and spontaneous Raman measurements (Figure 1i) at the C–D signature region, corresponding to the time‐lapse D_2_O cultured *P. aeruginosa*, reveal that D_2_O is incorporated into deuterated biomolecules inside the cell. The C–D abundance in single microbial cells increases as the D_2_O culture time. Moreover, both SRS and Raman spectra are peaked at 2160 cm^−1^, presumably corresponding to deuterated proteins or peptidoglycan which forms the cell wall.^[^ ^26^ ^]^ At 10 min, the stronger signal at the cell peripheral area is possibly due to rapid synthesis of peptidoglycan which is highly concentrated in cell wall. Collectively, our studies demonstrate that D_2_O incorporation into individual active microbial cells can be spatially and temporally visualized within 10 min by nondestructive SRS imaging at single bacterium level, and the amount of deuterium incorporation can be quantified reliably.

### 2.2 D_2_O Incorporation in the Presence of Antibiotics

**Figure 2:**
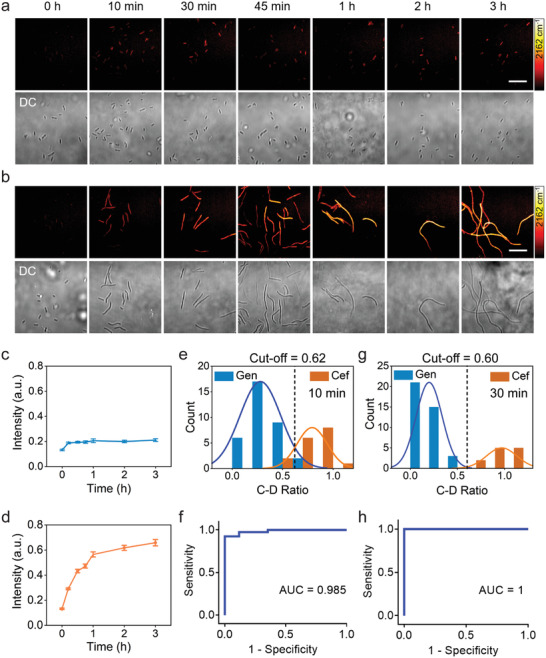
SRS‐based AST of *P. aeruginosa* as a function of culture time. Time lapse SRS at C–D and transmission images of *P. aeruginosa* after culture in D_2_O‐containing medium with the addition of 20 µg mL^−1^ gentamicin (a) or cefotaxime (b). Average C–D intensity plot over time for *P. aeruginosa* after culture in D_2_O‐containing medium with gentamicin (c) or cefotaxime (d) treatment. Number of cells *N* ≥ 10 per group. Error bars represent the SEM. Scale bar: 20 µm. Histogram plot of the count of bacteria as a function of C–D intensity ratio of antibiotic‐treated group over the control group after 10 min (e) and 30 min (g) treatment. ROC curves of 10 min (f) and 30 min (h) treatment illustrating the ability of the C–D intensity ratio to distinguish susceptible and resistant groups. AUC: area under the curve.

To examine how antibiotics affect the metabolic activity of D_2_O incorporation in bacteria, and to demonstrate that this effect can be used for rapid AST through SRS imaging, a cefotaxime‐resistant (MIC = 32 µg mL^−1^) and gentamicin‐susceptible (MIC = 4 µg mL^−1^) *P. aeruginosa* strain was selected as a model system. *P. aeruginosa* was cultured for different time in D_2_O containing medium, with 20 µg mL^−1^ gentamicin or cefotaxime. SRS imaging (**Figure** **2**a) and statistical analysis (Figure 2c) showed that C–D signals of bacteria were significantly reduced after culture with gentamicin, indicating inhibition of D_2_O incorporation in *P. aeruginosa* by gentamicin. On the contrary, Figure 2b,d shows that the C–D signals of *P. aeruginosa* cultured with cefotaxime increased with time, indicating active D_2_O incorporation in bacteria. We also observed that *P. aeruginosa* formed filaments after culture with cefotaxime (Figure 2b). This filamentary formation, which happens when Gram‐negative bacteria are treated with *β*‐lactam antibiotics, was also observed for *P. aeruginosa* treated with ceftazidime.^[^ ^10e^ ^]^ Notably, this filamentary formation might be incorrectly interpreted as growth by conventional imaging method.^[^ ^10e^ ^]^ Yet, it does not affect our metabolic activity measurements.

Next, we examined whether the rapid D_2_O incorporation inside bacteria can be used to differentiate the antimicrobial susceptibility. We used the relative C–D SRS intensity, the ratio between the antibiotic‐treated group and the antibiotic‐untreated group (Figure 1f), as a biomarker of antimicrobial susceptibility. To determine whether the SRS intensity ratio can be used to distinguish susceptible and resistant groups, the histogram of signal intensities for bacteria after 10 min culture was plotted over the intensity ratio (Figure 2e). The plots for susceptible and resistant groups were fitted with normal distribution. A cutoff at 0.62 was determined based on a 10 min culture of bacteria. The large area under curve (AUC = 0.985) in the corresponding receiver operating characteristic (ROC) curve plot clearly demonstrates the ability of this cutoff to separate the two groups (Figure 2f). These results indicate that our method is capable of determining susceptibility after 10 min D_2_O incubation time. The signal intensity ratio between the gentamicin‐susceptible and cefotaxime‐resistant groups showed significant difference at longer culture time (Figure S5, Supporting Information). A cutoff at 0.60 was obtained for the 30 min culture results (Figure 2g,h). In the following studies, we use 30 min of D_2_O incubation time to ensure sufficient signal to noise ratio and apply 0.60 cutoff to separate the metabolism active and inhibited conditions for bacteria cultured at different concentrations of antibiotics. In particular, we use such cutoff to define a single cell metabolism inhibition concentration (SC‐MIC) for a certain antibiotic: at or above SC‐MIC, the bacteria is susceptible and thus metabolically inactive; below SC‐MIC, the bacteria is resistant and thus metabolically active.

### 2.3 Quantitation of Susceptibility via SC‐MIC

**Figure 3:**
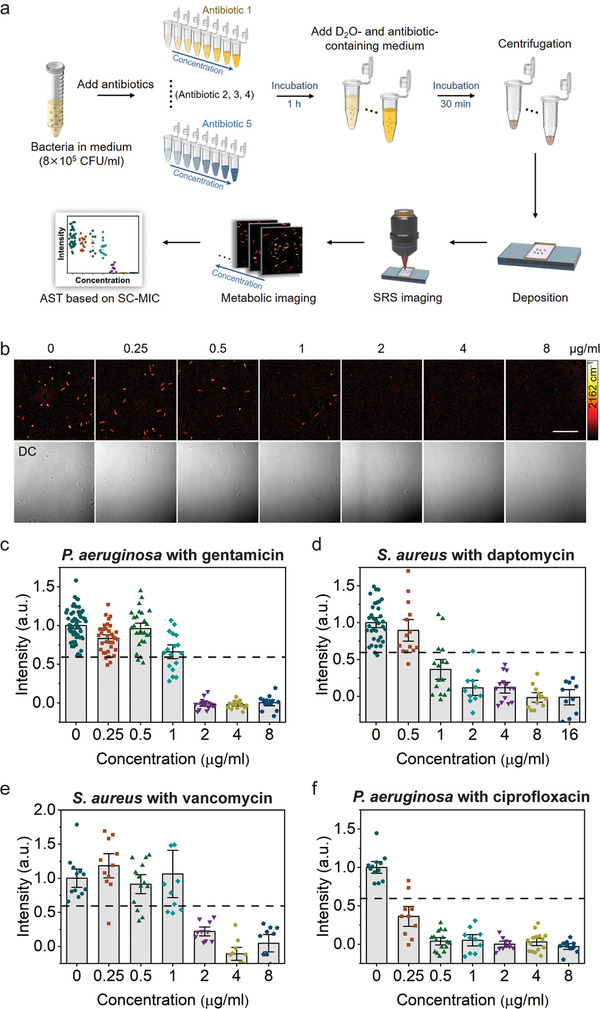
SC‐MIC determination via SRS imaging of D_2_O metabolic incorporation in single bacteria. a) Workflow of rapid AST with SC‐MIC determination by SRS imaging of D_2_O metabolic incorporation. b) SRS at C–D and corresponding transmission images of *P. aeruginosa* after culture in D_2_O containing medium with the addition of serially diluted gentamicin. c) Statistical analysis of C–D intensity in *P. aeruginosa* in (b). d–f) SC‐MIC determination for antibiotics with different mechanisms of action. The colored points under different concentration stand for different individual bacterium. The dotted lines indicate the cutoff value at 60% of the control sample. The C–D intensities are normalized to the mean of control without antibiotic treatment. Number of cells *N* ≥ 10 per group. Error bars represent the SEM. Scale bar: 20 µm.

To explore whether SRS imaging of D_2_O metabolic incorporation can quantify the response of bacteria to antibiotics and generate an SC‐MIC value comparable with the MIC, we tested *P. aeruginosa* with serially diluted gentamicin. Overnight cultured bacteria were diluted in cation‐adjusted Mueller–Hinton broth (MHB) medium to a final concentration of 8 × 10^5^ CFU mL^−1^. The bacteria were first treated with selected antibiotic containing medium for 1 h, then a medium containing D_2_O and the same concentration of antibiotics was added to bacteria for additional 30 min, in order to keep the final medium concentration and the final antibiotic concentration at the same level, meanwhile reaching a final D_2_O concentration of 70% (**Figure** **3**a). SRS imaging (Figure 3b) and statistical analysis (Figure 3c) showed that C–D signals at 2 µg mL^−1^ or higher gentamicin concentration were significantly lower than that in the control group (0 µg mL^−1^). With the previous determined threshold, D_2_O incorporation in *P. aeruginosa* was inhibited at 2 µg mL^−1^ and above concentrations. Therefore, the SC‐MIC was determined to be 2 µg mL^−1^. This value is within the onefold difference range with the MIC (4 µg mL^−1^) determined by the broth microdilution method. The statistical analysis of SC‐MIC determination for *Staphylococcus aureus* with daptomycin, *S. aureus* with vancomycin, *P. aeruginosa* with ciprofloxacin are shown in Figure 3e,f, respectively. The SC‐MIC results (1, 2, and 0.25 µg mL^−1^) match well with the MICs (1, 1, and ≤0.25 µg mL^−1^) by gold standard method, respectively. As shown in Figure 3e, under low concentration of antibiotic treatment, the C–D intensity increases a little, possibly due to the elevated stress responses and concurrently increased metabolic activity upon initial bactericidal antibiotic treatment.^[^ ^27^ ^]^ The colored points under different concentration stand for different individual bacterium. The intensity distribution comes from the metabolic heterogeneity of bacteria. The metabolic activities vary from cell to cell, particularly at different stages within one cell cycle.^[^ ^28^ ^]^ We note that some literatures studying D_2_O incorporation by spontaneous Raman spectroscopy have also observed the dispersive results of the bacterial metabolic activity, indicating the heterogeneity in the amount of incorporated deuterium among individual cells.^[^ ^19^, ^29^ ^]^ The C–D intensities upon antibiotic treatment are divided by the mean value of the control in each set of bacteria/antibiotic, and the cutoff value at 0.60 is shown by the dotted lines in Figure 3c–f. Taken together, we have developed a method to determine SC‐MIC that enables quantification of antimicrobial susceptibility.

### 2.4 SC‐MIC Measurement in 37 Sets of Samples

To validate the broad applicability of our method, we tested 8 major bacterial species and 14 different antibiotics often encountered in clinic (**Table** **1**). These antibiotics cover major bacterial inhibition mechanisms of action: inhibition of cell wall synthesis, protein synthesis, DNA synthesis, and/or cell membrane disruption. Typical SRS imaging (Figure S6, Supporting Information) and statistical analysis (Figure 3d–f) showed that antibiotics with all the mechanisms of action affect D_2_O incorporation in bacteria: the *β*‐lactam amoxicillin, the aminoglycoside gentamicin, the fluoroquinolone ciprofloxacin, and the cell membrane targeting daptomycin. We performed 37 sets of the experiments (Table 1), where SC‐MIC was obtained after 1.0 h incubation with antibiotics and additional 0.5 h incubation with D_2_O and antibiotics. For each set, the SC‐MIC determination by quantifying the SRS signal intensities versus the concentration of antibiotics using the cutoff value of 0.60 is presented as a heatmap. Table 1 shows that most MIC values matched well with the SC‐MICs when we select 0.60 as the cutoff. We note that some profiles of C–D intensities as a function of the antibiotic concentration show a sharp drop at a certain antibiotic concentration, meaning a sensitive response to antibiotics, or a flat trend upon the antibiotic treatment which means antibiotic‐resistant responding. Under these cases, the cutoff value 0.60 is valid to determine the SC‐MICs. Table 1 presents the SC‐MIC, MIC and the defined susceptibility category which is interpreted as “susceptible,” “resistant,” or “intermediate” according to the Clinical and Laboratory Standards Institute (CLSI) criteria for each tested bacterial strain. As compared with MIC determined by conventional broth microdilution assay, the SC‐MIC (highlighted in black boxes in Table 1) achieved a category agreement of 94.6% (35 out of 37), with 5.4% minor error (2 out of 37), no major error, and no very major error. These results satisfy US Food and Drug Administration (FDA) requirements for AST systems. Most of the SC‐MIC results were obtained after 1 h culture in antibiotic containing medium followed by 0.5 h culture in D_2_O and antibiotics‐containing medium. We observed that methicillin‐resistant *S. aureus* (MRSA) grew slower than susceptible *S. aureus*. Therefore, MRSA strains were cultured in D_2_O medium for 1 h to achieve comparable C–D signals. With automated imaging and data analysis (Figure S7, Supporting Information), the whole procedure from colony to results took less than 2.5 h for most of the bacterial strains tested, and 3 h for MRSA strains. Collectively, these results validate SRS imaging of D_2_O metabolic incorporation as a rapid and accurate AST method.

**Table 1:** Visualization of SC‐MIC results determined from SRS imaging of D_2_O metabolic incorporation after 1.5 h incubation time and comparison with MICs determined from gold standard broth microdilution method. The normalized intensity is calculated by normalizing the C–D intensities divided by the mean value of the control in each set of bacteria/antibiotic. The data are shown as the mean of C−D intensities measured with number of cells *N* ≥ 10 per group. The SC‐MICs determined by cutoff at 0.60 are highlighted with black boxes in the heatmap. The comparison of SC‐MIC and the CLSI classifications (interpreted as “susceptible,” “resistant,” or “intermediate”) of each strain based on broth microdilution is shown. VAN: vancomycin; LNZ: linezolid; DAP: daptomycin; GEN: gentamicin; ERY: erythromycin; TMP/SMX: trimethoprim/sulfamethoxazole; AMI: Amikacin; CIP: ciprofloxacin; DOX: doxycycline; TOB: tobramycin; IMI: imipenem; CTX: cefotaxime; AMO: amoxicillin; CL: colistin; S: sensitive; R: resistant; I: Intermediate

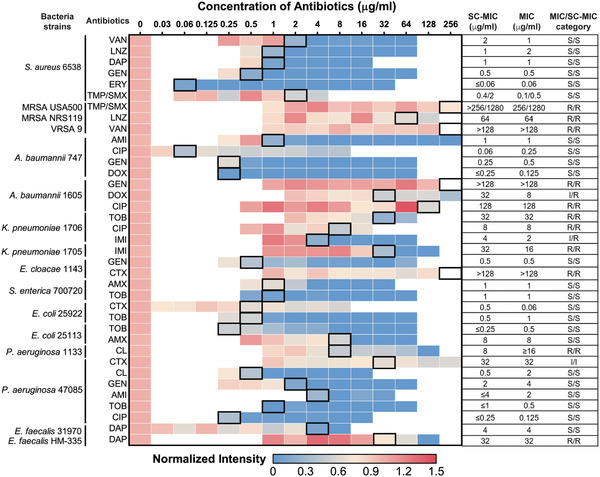

We further analyzed the SC‐MIC results in the 37 sets of samples. The 2 minor errors were both from Gram‐negative bacteria, resulting in a category agreement of 100% (11 of 11) for Gram‐positive samples (nine *S. aureus* samples and two *E. faecalis* samples), and a category agreement of 92.3% (24 of 26) with 7.7% minor error (2 of 26) for Gram‐negative samples. Though the category agreement in Gram‐negative bacterial strains was lower than that in Gram‐positive strains, these results still meet the FDA requirements (category agreement ≥90%, minor error ≤10.0%, major error ≤3.0%, very major error ≤1.5%).

As shown in Table 1, 32 SC‐MICs are identical or have onefold difference with MIC results, resulting in an essential agreement of 86.5% (32 of 37). Four SC‐MIC results have threefold difference, and one result has more than threefold difference. To better understand the good agreement and the residual discrepancy between SC‐MICs and MICs in these specimens, we obtained MICs of the 37 sets of samples by conventional broth microdilution assay in a blinded manner, using 70% D_2_O MHB as the culture medium. The results are listed in Table S1 (Supporting Information). Most of the MICs determined in 70% D_2_O MHB are identical or show only onefold difference with the MICs in normal MHB. Interestingly, for the five results that had the most differences between MIC and SC‐MIC, the MICs determined in 70% D_2_O MHB agreed more with SC‐MICs than MICs determined in normal MHB. Specifically, when *P. aeruginosa* was treated with colistin, a polypeptide that targets bacterial cell membrane, the SC‐MIC values were much lower than the MICs in normal MHB, but had much smaller difference from the MICs in 70% D_2_O MHB. This comparison indicates that 70% D_2_O might increase the vulnerability of some bacteria to certain antibiotics. Our sample preparation procedure is composed of two steps. In the first step, we treated bacteria with selected antibiotic containing medium for 1 h. This step was conducted in H_2_O solution and was the main step to influence bacterial metabolic activity upon antibiotic exposure. Then, in the second step, a medium containing D_2_O and the same concentration of antibiotics were added to bacteria for additional 30 min, in order to keep the final medium concentration and the final antibiotic concentration at the same level, meanwhile reaching a final D_2_O concentration of 70%. The second step aimed to use D_2_O to interrogate this system to study the metabolic response of bacteria. Overall, our SC‐MIC results were determined by the antimicrobial activity influence in normal medium for 1 h and the D_2_O incorporation for additional 30 min. This design minimized the impact of D_2_O influence of antimicrobial activity. As a result, we observed a good agreement of the MIC values for a large part of the targets shown in Table S1 (Supporting Information).

### 2.5 SC‐MIC for Bacteria in Urine Environment

**Figure 4:**
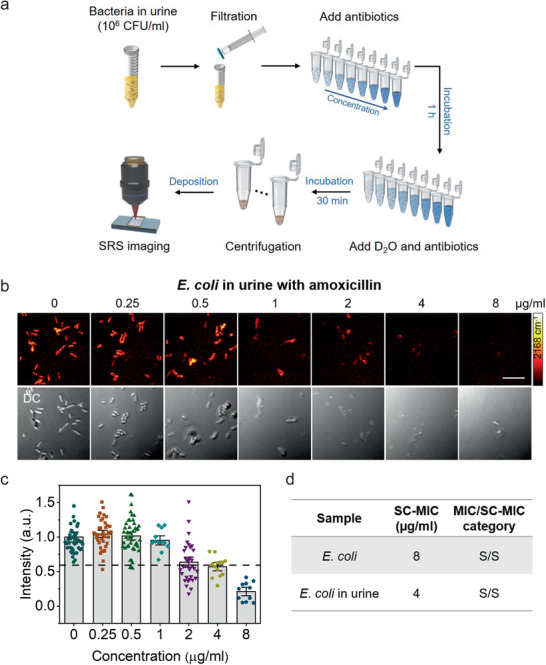
SC‐MIC determination after 1 h culture of *E. coli* in urine. a) Bacterial purification protocol for bacteria in urine for rapid AST by SRS imaging of D_2_O metabolic incorporation. b) SRS and corresponding transmission images of *E. coli* in urine after 1 h culture in D_2_O‐containing medium with the addition of serially diluted amoxicillin. c) Statistical analysis of C–D intensity in bacteria in (b). Number of cells *N* ≥ 10 per group. The colored points under different concentration stand for different individual bacterium. The dotted lines indicate the cutoff value at 60% of the control sample. The C–D intensities are normalized to the mean of control without antibiotic treatment. Number of cells *N* ≥ 10 per group. Error bars represent the SEM. Scale bar: 10 µm. d) The comparison of SC‐MIC and the CLSI susceptibility category for *E. coli* isolate and *E. coli* in urine. S: sensitive.

To investigate the potential of rapid AST by SRS imaging of D_2_O metabolic incorporation for clinical applications, we first tested bacteria in urine. For bacteria in urine, we tested *Escherichia coli*, which is the most common pathogen in urinary tract infection (UTI).^[^ ^30^ ^]^ To mimic the clinical UTI samples, we used spiked samples by adding *E. coli* to the urine at a final concentration of 10^6^ CFU mL^−1^. After filtration with 5 µm filter and centrifugation, the purified bacteria were used for SC‐MIC measurements (**Figure** **4**a). This sample preparation procedure took about 15 min. The clean background in SRS images showed that this convenient sample preparation procedure was favorable for rapid AST (Figure 4b). SC‐MIC for *E. coli* in urine with amoxicillin was determined to be 4 µg mL^−1^ (Figure 4b,c), which has the same essential and category agreement with the SC‐MIC or MIC for pure *E. coli* (Figure 4d). These results showed that rapid AST by SRS imaging of D_2_O metabolic incorporation is suitable for clinical application to bacteria in the urine.

### 2.6 SC‐MIC for Bacteria in Blood Environment

**Figure 5:**
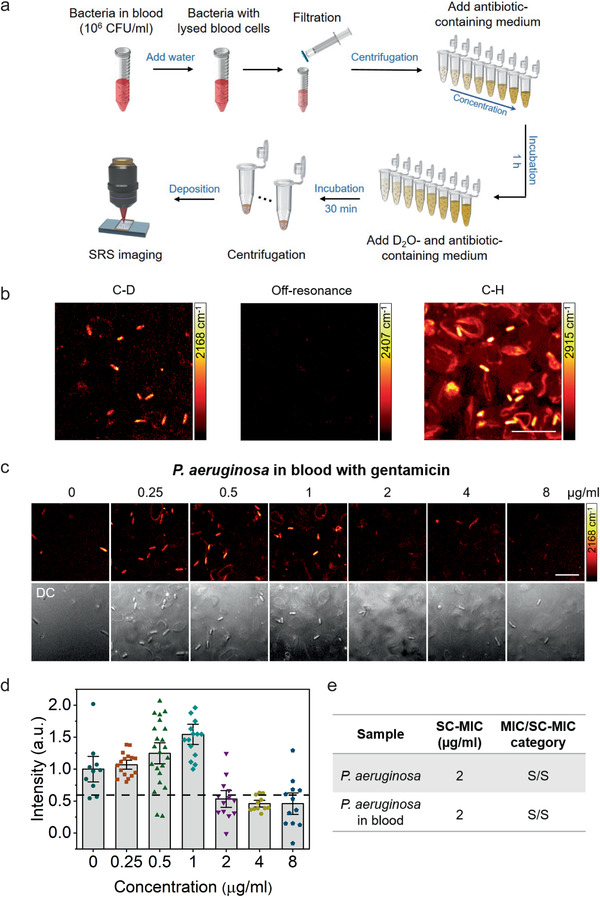
SC‐MIC determination after 1 h culture of *P. aeruginosa* in blood. a) Bacterial purification protocol for bacteria in blood for rapid AST by SRS imaging of D_2_O metabolic incorporation. b) SRS images at C–D, off‐resonance (2407 cm^−1^), and C–H of bacteria in blood after 1 h culture in D_2_O containing medium. c) SRS and corresponding transmission images of *P. aeruginosa* in blood after 1 h culture in D_2_O‐containing medium with the addition of serially diluted gentamicin. d) Statistical analysis of C–D intensity in bacteria in (c). The colored points under different concentration stand for different individual bacterium. The dotted lines indicate the cutoff value at 60% of the control sample. The C–D intensities are normalized to the mean of control without antibiotic treatment. Number of cells *N* ≥ 10 per group. Error bars represent the SEM. Scale bar: 10 µm. e) Comparison of SC‐MIC and susceptibility category for *P. aeruginosa* isolate and *P. aeruginosa* in blood. S: sensitive.

As compared with urine, blood includes a lot of blood cells and presents a much bigger challenge for in situ analysis of bacterial activity. To investigate the potential of rapid AST by SRS imaging of D_2_O metabolic incorporation for clinical bloodstream infections samples, we tested *P. aeruginosa* spiked in human blood. Bacteria were first added to blood at a final concentration of ≈10^6^ CFU mL^−1^ (**Figure** **5**a). Then, water was added to the mixture to lyse the blood cells. After filtration and centrifugation, the purified bacterial samples were used for SC‐MIC measurements. The whole procedure for sample preparation took about 15 min. After culture in D_2_O medium, SRS images at the C–H vibration showed a lot of debris or blood cells still left in the purified bacterial samples (Figure 5b). While, in the same area, the SRS image of C–D vibration was dominated by bacterial signal. The reason is that debris or red blood cells do not have metabolic activity to incorporate D_2_O unlike live bacteria. The weak background mostly comes from the cross‐phase modulation or photothermal signal of interferent species, which does not affect the quantification of SC‐MICs. The off‐resonance SRS images further confirmed that the signals in bacteria largely came from the C–D vibration (Figure 5b). The SC‐MIC value for *P. aeruginosa* in blood after 1 h culture was determined to be 2 µg mL^−1^ (Figure 5c,d), which agreed with the SC‐MIC or MIC for *P. aeruginosa* in growth medium (Figure 5e). These results collectively showed that SRS imaging of D_2_O metabolic incorporation can rapidly determine SC‐MIC for bacteria in blood environment.

We note that bacterial concentration in the spiked urine and blood samples was 10^6^ CFU mL^−1^ in our tests (Figures 4a and Figure 5a). Since clinically positive UTI samples usually contain more than 10^5^ CFU mL^−1^ of bacteria, the bacterial concentration can easily reach 10^6^ CFU mL^−1^ or higher after centrifugation.^[^ ^8a^ ^]^ The bacterial concentrations in positive blood cultures range from 10^6^ to 10^9^ CFU mL^−1^.^[^ ^31^ ^]^ Therefore, our SC‐MIC measurement can be directly used for UTI or positive blood culture samples.

## 3 Discussion

The current work demonstrates a rapid platform that can determine the susceptibility of bacteria in cation‐adjusted MHB medium, urine and blood by SRS imaging of D_2_O incorporation at a single bacterium level. Metabolic incorporation of D_2_O, which is used for biomolecule synthesis, was monitored in a single bacterium by SRS imaging of C–D bonds. Metabolic response was probed in as short as 10 min after culture in D_2_O medium. The total AST assay time from sample to susceptibility test is about 2 h, with the value of SC‐MIC obtained in less than 2.5 h from colony to results. The SC‐MIC results of 37 sets of bacterial isolate samples, which included 8 major bacterial species and 14 different antibiotics, were systematically studied and validated by MIC determined by the broth microdilution method, with a category agreement of 94.6% and 5.4% minor error. Furthermore, we investigated the feasibility of our method to study samples in complex biological environments. The SC‐MIC can be determined after 1 h culture of bacteria in urine and blood, which is considered a tremendous reduction in analysis time as compared with the conventional broth microdilution method.

Previously, we monitored the metabolic incorporation of glucose‐d_7_ in isolated bacteria or fungi using SRS microscopy.^[^ ^23^, ^32^ ^]^ Though glucose is the preferred carbon source for most bacterial growth,^[^ ^33^ ^]^ glucose‐d_7_ itself contains C–D bonds, which causes a background in the SRS image. In contrast, a major advantage of the D_2_O metabolism approach is the enabling of background‐free SRS imaging of bacterial metabolic activity in a complex environment such as whole blood, which is difficult with the glucose‐d_7_ approach. Specifically, incorporation of D atom into the newly synthesized lipids, proteins, and nucleic acids generates C–D bond,^[^ ^24^, ^34^ ^]^ of which the Raman peak resides in a silent spectral region, enabling sensitive and specific detection. In the present study, we monitored the D_2_O metabolic incorporation by tracking the speed and amount of C–D bond formation. Significantly, the C–D vibration is spectrally separated from the O–D vibration in D_2_O, allowing for background‐free SRS measurements. The second key advantage of D_2_O versus glucose is on the medium. In the previous work, we conducted the antimicrobial susceptibility test by evaluating the bacterial glucose‐d_7_ metabolic activity in glucose‐free M9 minimal medium. For generally used glucose‐containing LB or MHB medium, glucose‐d_7_ becomes a competitive carbon source, causing problems for quantitative study. In this work, we studied D_2_O metabolic activity in MHB, a standard medium used by gold standard broth dilution AST, enabling AST of a wide variety of bacteria species for comparison with the CLSI. Another innovation of this study is the use of femtosecond pulses, which significantly increased the signal to noise ratio and the imaging speed accordingly.

It is known that stationary‐phase and nondividing bacteria are common in many persistent infections (e.g., endocarditis and osteomyelitis) and in biofilm‐associated infectious diseases (e.g., periodontitis and cystic fibrosis).^[^ ^35^ ^]^ To evaluate the potential of our SRS metabolic imaging method for nondividing bacteria, we investigated the metabolic dynamics of D_2_O incorporation in *E. coli* starting from different phases, lag, log, and stationary phase (Figure S8, Supporting Information). Interestingly, we observed similar metabolic dynamics during the same period of time, which is consistent with the growth curves with optical density measurements (Figure S8d,e, Supporting Information). Hence, our SRS metabolic imaging measurement can be potentially applied to determine the susceptibility of bacteria at the predivision stage, which is beyond the reach of conventional culture method. Because NADPH is ubiquitously used in cell metabolism, our SRS metabolic imaging method has the potential of being broadly used for rapid AST in various strains and can be extended to determine the susceptibility in fungal infections. Another exciting application of this method is for slowly growing bacteria, like *Mycobacterium tuberculosis* which doubles roughly once per day and has a remarkably slow growth rate.^[^ ^36^ ^]^

A few groups reported coherent Raman imaging of D_2_O activity inside mammalians. Potma et al. used CARS microscopy to monitor D_2_O entry into a cell in real time.^[^ ^37^ ^]^ Shi et al. demonstrated picosecond SRS imaging of D_2_O metabolism in mammalian cells after 1 day incubation and in live animals after at least 2 day treatment.^[^ ^24a^ ^]^ Compared with mammalian cells, imaging D_2_O metabolic activity in a micrometer‐sized bacterium is challenging. Here, we deployed a few strategies to achieve good signal to noise ratio in a single scan. First, stimulated Raman loss is measured, where most excitation power in on the Stokes beam to minimize photodamage to the specimen. Second, femtosecond pulses are used for excitation of the broad C–D vibrational bands, which improved the signal to noise ratio by five times compared with picosecond pulses. Third, the cross‐phase modulation background is minimized by placing the bacteria on a poly‐l‐lysine‐coated glass substrate and covered with phosphate‐buffered saline solution.

For clinical specimens, each sample requires hours of preincubation to obtain bacterial isolates. Methods based on nanoliter array,^[^ ^8a^ ^]^ digital nucleic acid quantification,^[^ ^12b^ ^]^ and Raman spectroscopy^[^ ^14^ ^]^ have been developed to perform AST for clinical urine samples. Compared with urinary tract infections, bloodstream infections or sepsis are more life‐threatening cases,^[^ ^12^, ^38^ ^]^ where rapid AST is urgently needed. A direct AST method using microscopic imaging of bacterial colony formation can identify appropriate antimicrobial agents and provide MIC results from positive blood cultures.^[^ ^17^ ^]^ However, this method relies on bacterial growth and takes at least 6 h to perform AST. Commercial automatic systems^[^ ^39^ ^]^ and mass spectrometry^[^ ^40^ ^]^ strategies allow for direct AST from positive blood cultures, but they could not serve to provide the MIC results for clinical decision. In this work, we demonstrate in situ SRS imaging of D_2_O metabolic incorporation in single bacteria at a clinically relevant concentration (10^5^–10^6^ CFU mL^−1^) in either urine or whole blood. This capacity paves the way toward clinical translation of our technology. While we need to know the antimicrobial susceptibility of bacteria, MIC determination is even more significant in clinics to avoid excess dosage of antibiotics to patients to cause potential side effects.^[^ ^41^ ^]^ We would emphasize that our SC‐MIC method is capable of detecting MICs and susceptibility classification for each strain/antibiotic. Compared with the spontaneous Raman microscopy, our method requires tremendously reduced data acquisition time (≈600 times less) to obtain MIC results due to orders‐of‐magnitude signal enhancement. Based on our method, the MICs are determined after 1 h antibiotic treatment and 30 min mixture of D_2_O and antibiotics incubation into bacteria in urine and blood. Each SRS image, containing at least 10 bacteria, was acquired within ≈1 s in one single shot, while it takes about 10 min by spontaneous Raman measurement. We estimate the total MIC assay time (excluding the manually operation time) to study ten antibiotics per strain/antibiotic set is less than 2.5 h from sample to MIC results, which is much more efficient and competitive in determining MICs. In one batch of experiment, we actually prepared in parallel 40 samples corresponding to treatment by five different antibiotics, each with eight concentrations. High throughput measurement is possible because each SRS images takes ≈1 s. However, because the samples are handled manually, the time for measuring five different antibiotics is longer than 2.5 h. In future work, we will employ automated sample preparation and data acquisition in a multi‐well chamber to further improve the throughput. We have compared our method with other nonisolation based AST techniques in Table S3 (Supporting Information).

Finally, given the importance of identification of pathogens in clinical decision‐making, our SRS metabolic imaging can be integrated with diagnostic methods that are capable of rapid identification of pathogens, for example, matrix‐assisted laser desorption ionization‐time‐of‐flight mass spectrometry.^[^ ^31^, ^40^, ^42^ ^]^ Integration of these in situ analysis tools and translation into clinic could potentially eliminate the “culture to colony” paradigm, thus allowing for on‐time identification of appropriate antimicrobial agents for precise treatment.

## Conflict of Interest

The authors declare no conflict of interest.

## Author Contributions

M.Z. and W.H. contributed equally to this work. J.‐X.C and W.H. conceived the idea. J.‐X.C., W.H., M.Z., M.S., and P.W. designed the experiments. M.Z., W.H., N.S.A., J.L., P.‐T.D., and C.Z. conducted the experiments and analyzed the data. M.Z., W.H., and J.‐X.C. cowrote the manuscript. All authors have contributed to discussing and editing the manuscript, and given approval to the final version of the manuscript.

## Supporting information

Supporting Information
