# Supplementary material for: Rapid Determination of Antimicrobial Susceptibility by Stimulated Raman Scattering Imaging of D2O Metabolic Incorporation in a Single Bacterium
Source: Adv Sci (Weinh). 2020 Aug 16;7(19):2001452. doi: 10.1002/advs.202001452 (PMC7539191; doi:10.1002/advs.202001452)
Supplement: Supplementary file 1 — Supporting Information [file ADVS-7-2001452-s001.pdf]

© 2020 Wiley-VCH GmbH

ADVANCED SCIENCE
Open Access

# Supporting Information

for *Adv. Sci.,* DOI: 10.1002/advs.202001452

# Rapid Determination of Antimicrobial Susceptibility by Stimulated Raman Scattering Imaging of $D_2O$ Metabolic Incorporation in a Single Bacterium

*Meng Zhang[#], Weili Hong*[,#], Nader S. Abutaleb, Junjie Li, Pu-Ting Dong, Cheng Zong, Pu Wang, Mohamed N. Seleem, Ji-Xin Cheng**

# Supporting Information

**Rapid Determination of Antimicrobial Susceptibility by Stimulated Raman Scattering Imaging of $D_2O$ Metabolic Incorporation in a Single Bacterium**

*Meng Zhang[#], Weili Hong*[,#], Nader S. Abutaleb, Junjie Li, Pu-Ting Dong, Cheng Zong, Pu Wang, Mohamed N. Seleem, Ji-Xin Cheng**

*Correspondence to Ji-Xin Cheng (jxcheng@bu.edu) and Weili Hong (wlhong@vibronixinc.com).

# These authors contributed equally.

**This file includes:**
Materials and Methods
Figures S1 to S8
Tables S1 to S3

**Materials and Methods**

**SRS microscope.** A femtosecond (fs) pulsed laser (InSight DeepSee, Spectra-Physics) with an 80-MHz repetition rate and dual outputs was employed for excitation. One 120 fs laser with tunable 680−1100 nm wavelength served as the pump beam. The other 220 fs laser, centered at 1040 nm wavelength and served as the Stokes beam, was modulated by an acousto-optical modulator (AOM, 1205-C, Isomet) at ~2.4 MHz. The two beams were collinearly combined through a dichroic mirror. When spectral focusing is needed for hyperspectral SRS, both beams were chirped with two 15 cm long SF57 glass rods. After chirping, the pulse durations of the pump and Stokes laser were 1.9 ps and 1.3 ps, respectively. For implementation of SRS imaging with femtosecond pulses, the glass rods were removed from the path. The pump and Stokes beams were directed into a lab-built laser scanning microscope with a 2D galvo mirror for laser scanning. A 60× water objective (NA=1.2, UPlanApo/IR, Olympus) was used to focus the lasers to the sample, and an oil condenser (NA=1.4, U-AAC, Olympus) was used to collect the signal from the sample. Two filters (HQ825/150m, Chroma) were used to filter out the Stokes beam, the pump beam was detected by a photodiode (S3994-01, Hamamatsu) and the stimulated Raman signal was extracted by a lock-in amplifier (HF2LI, Zurich Instrument).

To image bacteria at the C-D vibrational frequency, the pump wavelength was tuned to 852 nm, and the power at the sample was ~8 mW; the Stokes power at the sample was ~40 mW. Each image contains 200 × 200 pixels and the pixel dwell time is 30 µs, resulting in an image acquisition time of ~1.2 seconds. Hyperspectral SRS imaging was conducted by temporally tuning the delay of the chirped pump and Stokes beams.

**Biological Specimens.** Bacterial strains used in this study (**Table S2**) were obtained from the Biodefense and Emerging Infections Research Resources Repository (BEI Resources) and the

American Type Culture Collection (ATCC). AST study of deidentified urine and deidentified whole blood was achieved by measuring the bacteria-spiked samples.

**Sample preparation.** To make $D_2O$ containing Lauria-Bertani broth (LB) (Sigma Aldrich) or cation-adjusted cation-adjusted Mueller-Hinton Broth (MHB) (Thermo Fisher Scientific) media, $D_2O$ was first mixed with purified water, then LB or MHB powder was added to the solution. The solution was sterilized by filtering. To prepare bacterial samples for SRS imaging, bacterial strains were cultured in normal medium to reach the logarithmic phase, then bacteria were diluted to ~$10^6$ CFU/ml in the $D_2O$-containing medium. After a controlled culture time, 500 µl sample was centrifuged, washed twice with purified water, fixed by 10% (w/v) formalin solution (Thermo Fisher Scientific) and deposited to an agarose gel pad or poly-L-lysine coated coverglasses. To prepare samples for spontaneous Raman spectroscopy, bacteria were cultured in normal or 70% $D_2O$-containing medium for 2 h, and then centrifuged and washed twice with purified water to remove the culture medium. For the time lapse experiment, *P. aeruginosa* ATCC 47085 were cultured in 70% $D_2O$ containing medium for up to 3 h. At different time points, 500 µl bacteria were centrifuged, washed twice with purified water, and deposited on a poly-L-lysine coated slide for imaging.

**$D_2O$ toxicity measurement.** $D_2O$ toxicity measurement was done in 96-well plates. *P. aeruginosa* ATCC 47085, *E. coli* BW25113, or *S. aureus* ATCC6538were cultivated into $D_2O$-containing LB medium with $D_2O$ concentrations ranging from 0 to 100%, and incubated at 37 °C. At each time point, optical densities (OD) of the samples were measured at 600 nm wavelength.

**Image processing and data acquisition**. To obtain the average C-D signal intensity, SRS images were processed with ImageJ software (ImageJ, NIH) (Figure S7). Briefly, SRS images

opened in ImageJ were first converted into 8-bit type images with inverted color for the following process. Then, the images were filtered with Gaussian blur, followed by image threshold adjustment to select bacterial area. The threshold was adjusted so the selected bacterial sizes matched those in the original SRS images. Next, particles analysis was applied to label and determine the area of bacteria. For bacterial determination, small particles were eliminated by adjusting the size threshold to determine the particles. By applying the same label and area of bacteria to the original SRS images, the average intensity of each data point was determined after subtraction of the background.

**Spontaneous Raman spectroscopy.** Bacteria in solution were deposited on a coverglass for spontaneous Raman measurement. Spontaneous Raman spectra of bacteria were acquired with an inverted Raman spectrometer (LabRAM HR evolution, Horiba scientific) with 532 nm laser source. The laser power at the sample was ~12 mW after a 40× air objective, and the acqusition time was 10 s. The grating was 600 l/mm.

**MIC determination.** The minimum inhibitory concentrations (MICs) of the antibiotics were determined using the broth microdilution method, according to guidelines outlined by the Clinical and Laboratory Standards Institute (CLSI).[1] Bacterial strains were grown aerobically overnight on tryptone soya agar plates at 37° C, afterwards a bacterial solution was diluted in normal or 70% $D_2O$-containing cation adjusted Mueller-Hinton broth (CAMHB) (or CAMHB supplemented to 50 μg/ml calcium for daptomycin), to achieve a bacterial concentration of about $5\times10^5$ CFU/ml and seeded in 96-well well plates. Antibiotics were added in the first row of the 96-well plates and serially diluted along the plates. Plates were then incubated aerobically at 37° C for 18-20 hours. MICs reported in this work are the minimum concentration of the antibiotics that completely inhibited the visual growth of the bacteria. The MIC values were

interpreted according to the interpretive standards of the Clinical and Laboratory Standards Institute (CLSI).[2]

**Supporting Figures**

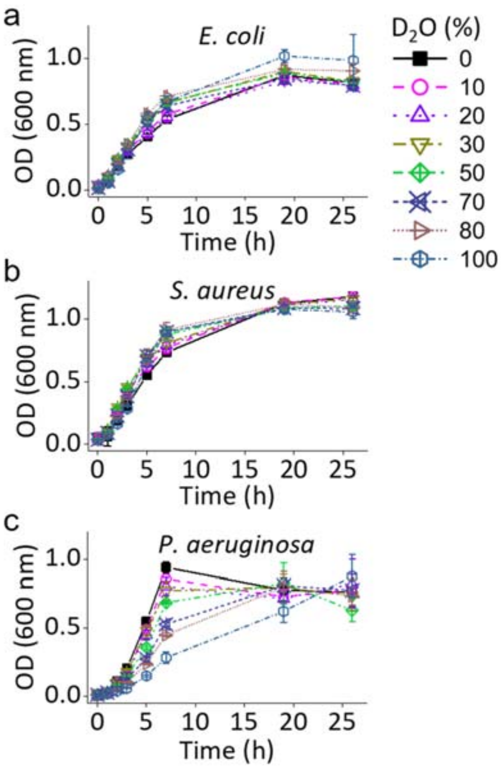


**Figure S1.** Testing $D_2O$ toxicity to bacterial growth. Growth curve of *E. coli*. (a), *S. aureus* (b) and *P. aeruginosa* (c) cultured in LB medium with different $D_2O$ concentrations with optical density (OD) measurements at 600 nm. Error bars indicate standard deviation values (number of measurements = 5).

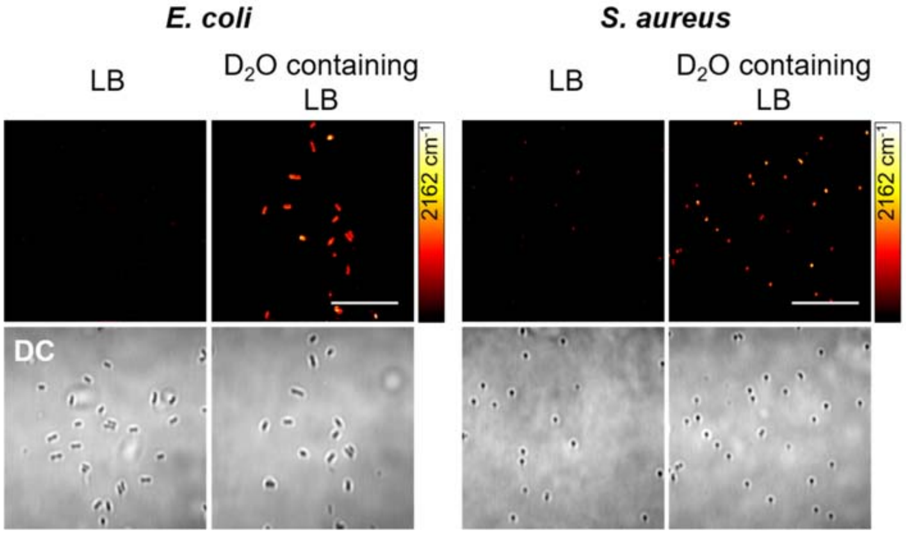


**Figure S2.** SRS and corresponding transmission images of *E. coli* and *S. aureus* after being cultured in LB and $D_2O$ containing LB medium for 3 h. Scale bar: 20 μm.

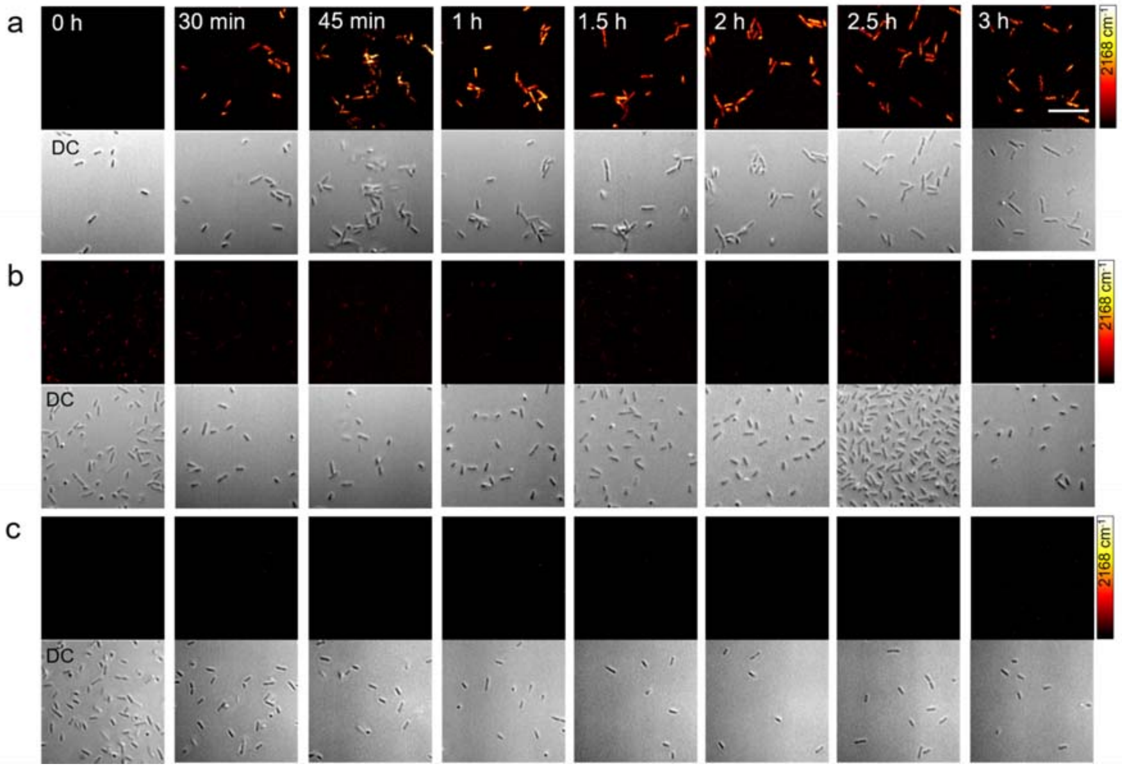


**Figure S3.** SRS imaging of $D_2O$ metabolic activity in (a) 37 °C live, (b) 4 °C live and (c) 37 °C fixed *P. aeruginosa* ATCC 47085 cultured in 70% $D_2O$ containing MHB medium with different incubation times. Scale bar: 10 μm.

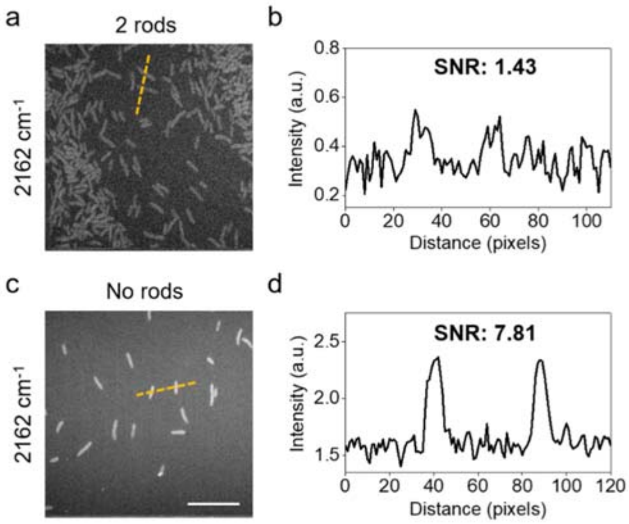


**Figure S4.** Femtosecond SRS improves signal to noise ratio (SNR) at C-D vibrational region over the chirped SRS. (a) SRS image at ~2162 $cm^{-1}$ of *P. aeruginosa* 47085 cultured in 70% $D_2O$ containing LB medium for 30 min with picosecond pulses generated by chirping with two SF57 glass rods. (b) Intensity plot of the orange line over bacteria in (a). (c) SRS image at ~2162 $cm^{-1}$ of *P. aeruginosa* cultured in 70% $D_2O$ containing LB medium for 30 min with femtosecond pulses. (d) Intensity plot of the orange line over bacteria in (c). Scale bar: 20 µm.

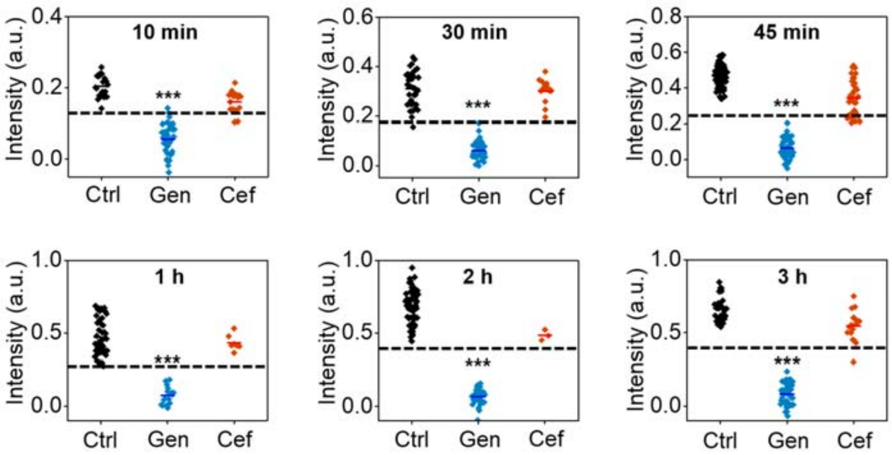


**Figure S5.** Statistical analysis of C-D intensity in *P aeruginosa* without treatment and with gentamicin or cefotaxime treatment for different times. Black bars indicate the threshold that discriminates susceptible (gentamicin) and resistant (cefotaxime) groups.

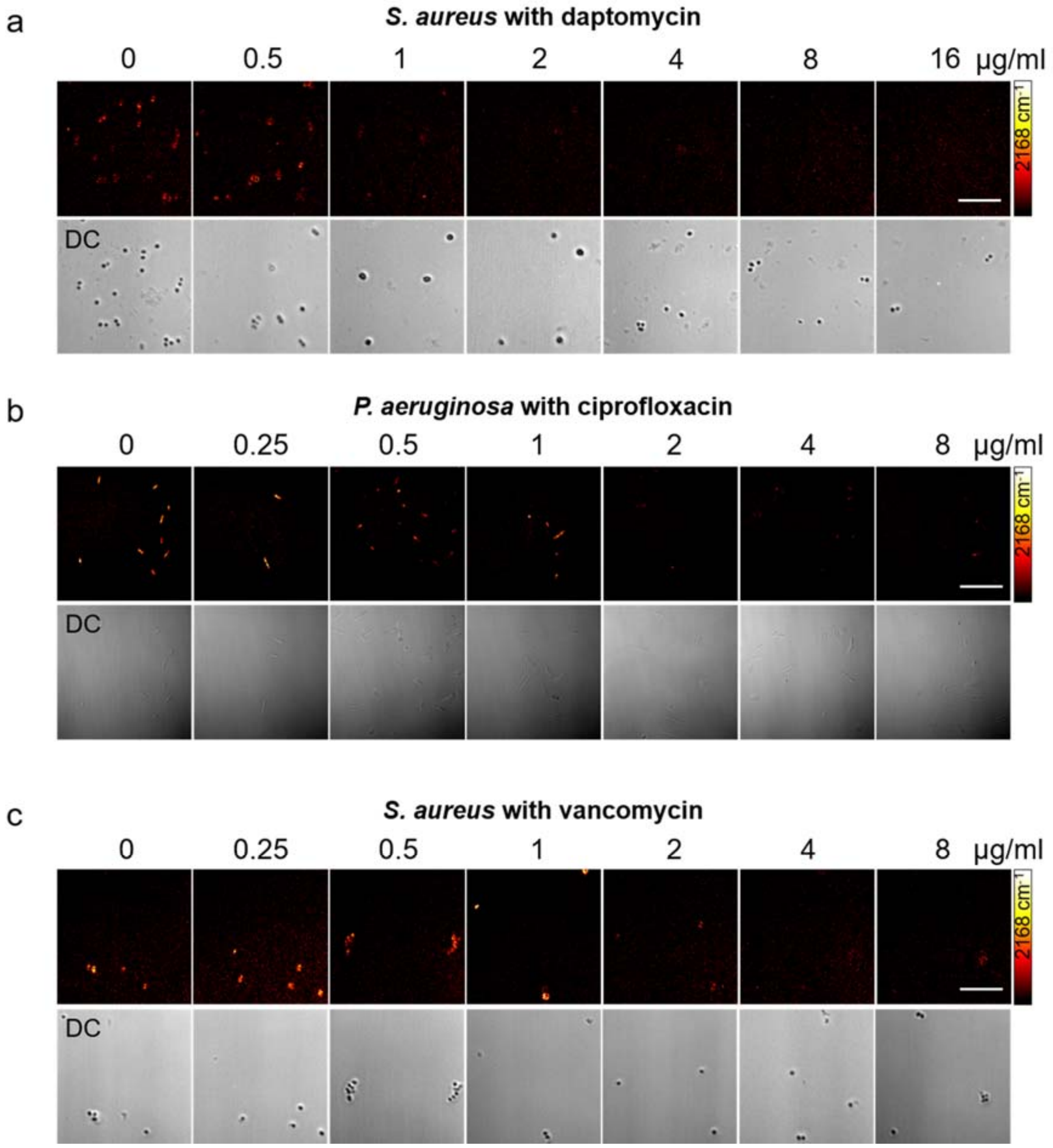


**Figure S6.** SC-MIC determination for *S. aureus* and *P. aeruginosa* treated with different mechanism of action. (a-c) SRS and corresponding images of bacteria cultivated in antibiotics for 30 min and $D_2O$ containing medium for another 30 min. Scale bar: (a, c) 10 µm; (b) 20 µm.

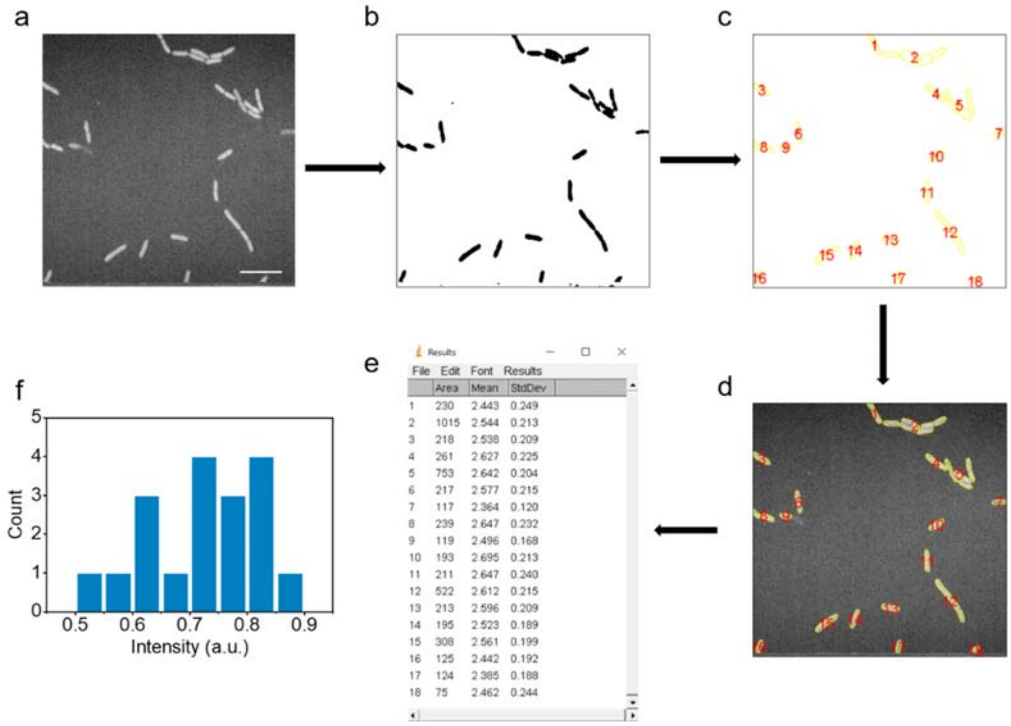


**Figure S7.** Automated image processing and data interpretation. (a) Original image. (b) Image after threshold adjustment to determine the area of bacterial cells. (c) Data points selected after particle analyze. (d) The corresponding data points selected in the raw image. (e) Results of the corresponding data points in the raw image. (f) Statistical results of the average intensity of the data points after subtraction of background. Scale bar: 10 μm.

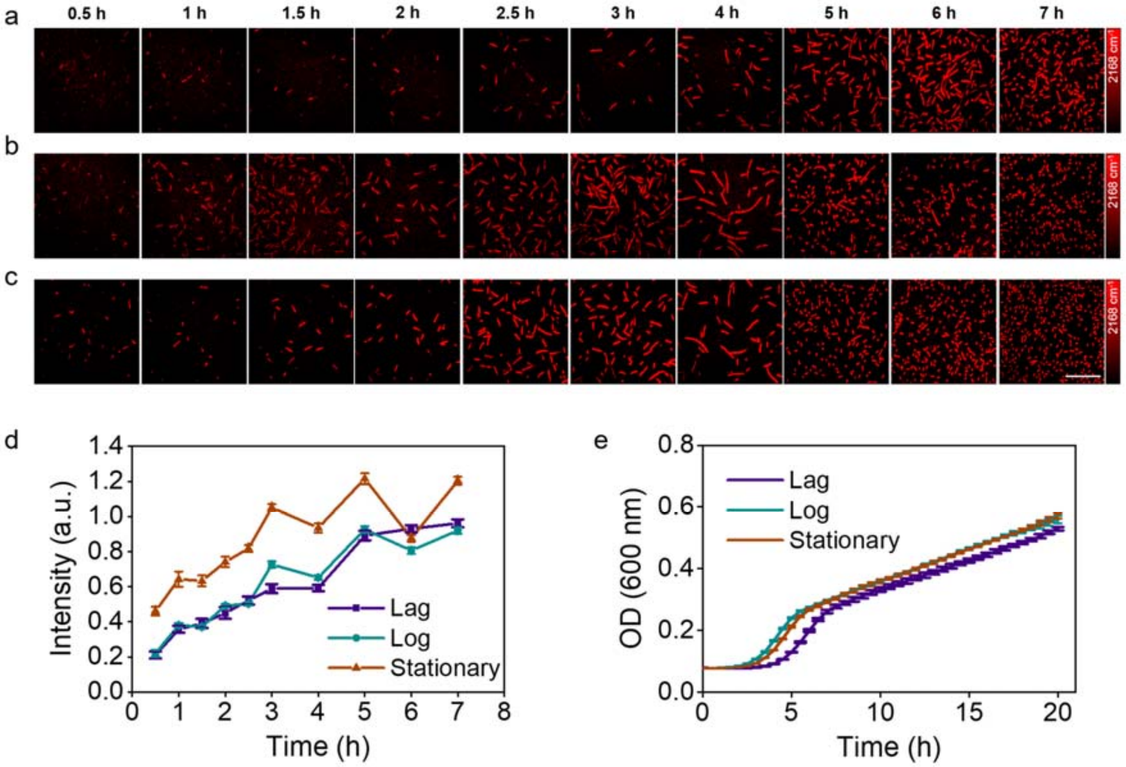


**Figure S8.** SRS imaging of $D_2O$ metabolic activity in *E. coli* BW 25113 from (a) lag phase, (b) log phase and (c) stationary phase cultured in 70% $D_2O$ MHB medium with different incubation time. Scale bar: 20 μm. (d) Average C-D intensity plot over incubation time for bacteria in (a) with $N \geq 10$ per group. (e) Growth curve of *E. coli* from the same batch as (a) with optical density (OD) measurements at 600 nm (number of measurements = 3). Error bars represent the standard deviation (SD).

**Table S1.** List of MICs obtained in normal MHB medium and in 70% $D_2O$-containing MHB medium.

| **Bacteria strains** | **Antibiotics** | **MIC in normal MHB (µg/ml)** | **MIC in 70% $D_2O$ MHB (µg/ml)** |
|---|---|---|---|
| *S. aureus* ATCC 6538 | Vancomycin | 1 | 0.5 |
| | Linezolid | 2 | 1 |
| | Daptomycin | 1 | 0.25 |
| | Gentamicin | 0.5 | 0.25 |
| | Erythromycin | 0.06 | 0.125 |
| | Trimethoprim/ Sulfamethoxazole | 0.1/0.5 | 0.1/0.5 |
| Methicillin-resistant *S. aureus* (MRSA) NRS 385 (MRSA USA500) | Trimethoprim/ Sulfamethoxazole | 256/1280 | >12.8/64 |
| Methicillin-resistant *S. aureus* (MRSA) NRS 119 | Linezolid | 64 | >64 |
| Vancomycin-resistant *S. aureus* (VRSA) NR-46419 (VRSA 9) | Vancomycin | >128 | >64 |
| *A. baumannii* ATCC BAA-747 | Amikacin | 1 | 0.5 |
| | Ciprofloxacin | 0.25 | 0.06 |
| | Gentamicin | 0.5 | 0.25 |
| | Doxycycline | 0.125 | 0.5 |
| *A. baumannii* ATCC BAA 1605 | Gentamicin | >128 | 64 |
| | Doxycycline | 8 | >64 |
| | Ciprofloxacin | 128 | 64 |
| *K. pneumoniae* ATCC BAA 1706 | Tobramycin | 32 | 32 |
| | Ciprofloxacin | 8 | 2 |
| | Imipenem | 2 | 1 |
| *K. pneumoniae* ATCC BAA 1705 | Imipenem | 16 | 16 |
| *E. cloacae* ATCC BAA 1143 | Gentamicin | 0.5 | 0.5 |
| | Cefotaxime | >128 | >64 |
| *S. enterica* ATCC 700720 | Amoxicillin | 1 | 1 |
| | Tobramycin | 1 | 1 |
| *E. coli* ATCC 25922 | Cefotaxime | 0.06 | 0.06 |
| | Tobramycin | 1 | 0.5 |
| *E. coli* BW 25113 | Tobramycin | 0.5 | 1 |
| | Amoxicillin | 8 | 4 |
| *P. aeruginosa* 1133 | Colistin | ≥16 | 32 |
| *P. aeruginosa* ATCC 47085 (PAO1) | Cefotaxime | 16 | 8 |
| | Colistin | 2 | 0.5 |
| | Gentamicin | 4 | 1 |
| | Amikacin | 2 | 2 |
| | Tobramycin | 0.5 | 0.5 |
| | Ciprofloxacin | 0.125 | 0.125 |
| *E. faecalis* NR-31970 | Daptomycin | 4 | 0.06 |
| *E. faecalis* HM-335 | Daptomycin | 32 | 32 |

**Table S2.** List of bacterial strains used in this study.

| **Bacterial strains** | **Source/ Description** |
|---|---|
| *S. aureus* ATCC 6538 | Quality control strain<br>Isolated from human lesion |
| Methicillin-resistant *S. aureus* (MRSA) NRS 385 (MRSA USA500) | Isolated from a bloodstream sample in Connecticut, USA. It is a hospital-acquired methicillin-resistant S. aureus (HA-MRSA) strain. |
| Methicillin-resistant *S. aureus* (MRSA) NRS 119 | Isolated in 2001 from an 85-year-old male with dialysis-associated peritonitis in Massachusetts, USA. |
| Vancomycin-resistant *S. aureus* (VRSA) NR-46419 (VRSA 9) | Isolated in 2007 in Michigan, USA from a left plantar foot wound of a 54-year-old female who recently received a 4-week course of vancomycin and levofloxacin to treat osteomyelitis of the left metatarsals |
| *A. baumannii* ATCC BAA-747 | Human clinical specimen<br>Isolated from ear pus<br>Quality control strain |
| *A. baumannii* ATCC BAA 1605 | Isolated from sputum of military personnel returning from Afghanistan entering a Canadian hospital, June 30, 2006<br>Resistant to Ceftazidime, Gentamicin, Ticarcillin, Piperacillin, Aztreonam, Cefepime, Ciprofloxacin, Imipenem, and Meropemem.<br>Sensitive to Amikacin and Tobramycin |
| *K. pneumoniae* ATCC BAA 1706 | Quality control strain |
| *K. pneumoniae* ATCC BAA 1705 | Isolated from a urine sample of 42-year-old male<br>*blaKPC* positive<br>Carbapenem-resistant (Imipenem and Ertrapenem) |
| *E. cloacae* ATCC BAA 1143 | A control strain, derived from an existing strain, 1982 |
| *S. enterica* ATCC 700720 | Wild-type strain isolated from a natural source, 1948<br>Used for: Emerging infectious disease research |
| *E. coli* ATCC 25922 | Quality control strain for antibiotics susceptibility testing |
| *E. coli* BW 25113 | The parent strain of the Keio collection comprising nearly 4,000 single-gene deletion mutants |
| *P. aeruginosa* 1133 | Quality control strain<br>Polymexin B- and colistin-resistant |
| *P. aeruginosa* ATCC 47085 (PAO1) | A laboratory strain constructed by stable integration of a mini-D3112 transposable element containing sequences allowing lacZalpha complementation<br>Used for transformation host and opportunistic pathogen research |
| *E. faecalis* NR-31970 | Isolated in 2001 from a urine sample in Michigan, USA.<br>Resistant to erythromycin and gentamicin |
| *E. faecalis* HM-335 | Isolated in 2004 from the blood of a 64-year-old female hemodialysis patient with fatal bacteremia after treatment with daptomycin.<br>Resistant to daptomycin |

Table S3. Comparison between existing non-isolation based AST techniques and our method.

| AST method | Mechanism | Time of sample preparation | Time for test | Sensitivity | Specificity | Cost for an apparatus | Cost for a single test | Direct on patient sample | Real MIC | Ref |
|---|---|---|---|---|---|---|---|---|---|---|
| **Stationary nanoliter droplet array** | Fluorescence dye labeling to quantify metabolism | 30-50 min | <5.5 h | Good/ 4 CFU per well | Good | Chip/fluorescence microscope | Low | Yes/Urine | No | 8a |
| **Digital real-time loop-mediated isothermal amplification** | DNA concentration | 15 min | 15 min | Good/ 700 copy in 6 μl | Good | Cost of reagent and LAMP | Low | Yes/Urine | No | 12b |
| **Light scattering microscopy** | Number of bacteria | Unprocessed patients' sample | 90 min | Single cell/ $10^7$ cells/ml | Good | Camera/laser | Low | Yes/Urine | No | 15 |
| **Diagnostic fidget spinner** | Colorimetric assay | 70 min | 50 min | $10^3$ -$10^6$ CFU/ml | Good | Low | Low | Yes/Urine | No | 16 |
| **Direct and rapid antimicrobial susceptibility testing** | Colony-forming area | | 6 h | $5.0\times10^5$-$5.0\times10^7$ CFU/ml | Good | Chip/Automated imaging | Low | Yes/Blood | Yes | 17 |
| **Spontaneous Raman** | Deuterium labeling to quantify metabolism | 1.5 h | 1 h | Low | Good | Raman microscope | Low | Yes/Urine | No | 14 |
| **SRS metabolic imaging** | Deuterium labeling to quantify metabolism | 2 h | <0.5 h | Good, at single-cell level | Good | SRS microscope | Low | Yes/Urine and Blood | Yes | This work |

## References


[1] Clinical and Laboratory Standards Institute (CLSI). Methods for Dilution Antimicrobial Susceptibility Tests for Bacteria That Grow Aerobically. 11th ed. CLSI standard M07. Wayne, PA: CLSI; 2018.

[2] Clinical and Laboratory Standards Institute (CLSI). Performance Standards for Antimicrobial Susceptibility Testing. 29th ed. CLSI supplement M100. Wayne, PA: CLSI; 2019.
